# An innovative anti-rotation tension band wiring for treating transverse patellar fractures: finite element analysis and mechanical testing

**DOI:** 10.1186/s13018-024-04902-w

**Published:** 2024-07-19

**Authors:** Ze Zhang, Fengpo Sun, Tongyi Zhang, Liangyuan Wen

**Affiliations:** Department of Orthopedics, Beijing Hospital, National Center of Gerontology, Institute of Geriatric Medicine, Chinese Academy of Medical Sciences & Peking Union Medical College, Beijing, P.R. China

**Keywords:** Patella fracture, K-wire, Rotation, FEA, Biomechanics test

## Abstract

**Background:**

The displacement and rotation of the Kirschner wire (K-wire) in the traditional tension band wiring (TBW) led to a high rate of postoperative complications. The anti-rotation tension band wiring (ARTBW) could address these issues and achieve satisfactory clinical outcomes. This study aimed to investigate the biomechanical performance of the ARTBW in treating transverse patellar fracture compared to traditional TBW using finite element analysis (FEA) and mechanical testing.

**Methods:**

We conducted a FEA to evaluate the biomechanical performance of traditional TBW and ARTBW at knee flexion angles of 20°, 45°, and 90°. Furthermore, we compared the mechanical properties under a 45° knee flexion through static tensile tests and dynamic fatigue testing. The K-wire pull-out test was also conducted to evaluate the bonding strength between K-wires and cancellous bone of two surgical approaches.

**Results:**

The outcome of FEA demonstrated the compression force on the articular surface of ARTBW was 28.11%, 27.32%, and 52.86% higher than traditional TBW at knee flexion angles of 20°, 45°, and 90°, respectively. In mechanical testing, the mechanical properties of ARTBW were similar to the traditional TBW. In the K-wire pull-out test, the pull-out strength of ARTBW was significantly greater than the traditional TBW (111.58 ± 2.38 N vs. 64.71 ± 4.22 N, *P* < 0.001).

**Conclusions:**

The ARTBW retained the advantages of traditional TBW, and achieved greater compression force of articular surface, and greater pull-out strength of K-wires. Moreover, ARTBW effectively avoided the rotation of the K-wires. Therefore, ARTBW demonstrates potential as a promising technique for treating patellar fractures.

**Supplementary Information:**

The online version contains supplementary material available at 10.1186/s13018-024-04902-w.

## Introduction

The patella is a critical component of the knee extensor mechanism, enhancing the strength of the quadriceps by approximately 30% [1, 2]. Patellar fractures are common lower limb injuries, accounting for approximately 1% of all skeletal injuries [3]. Surgical intervention was recommended for patellar fractures with a displacement exceeding 2 mm [2, 4].

Various surgical techniques, such as TBW, cannulated screw, cable-pin, angle-stable plate, and claw-like shape memory alloy, were used to apply in displaced patellar fracture [4–10]. During the knee flexion, the tension on the patellar anterior surface will converted into compression force on the articular surface [11]. Due to its distinctive biomechanical properties, stable fixation efficiency, and allowing for early mobilization, the TBW technique has become the most widely used surgical approach for patella fractures [4, 12–15]. Additionally, non-metallic fixation and orthobiologics have also emerged in the field of management of patellar fractures in recent years [16–18].

Lazaro et al. reported a rate of 37% hardware removal due to prominent and symptomatic implants as a result of breakage or continuous soft tissue irritation [4, 19]. Loss of reduction most commonly involved the loosening of TBW structure, with a rate of 20% [7, 20, 21]. The rotation and displacement of the K-wire may lead to symptomatic implants and loosening of TBW. Additionally, the rate of ulceration attributed to the rotation of K-wires reached up to 40% [22]. Therefore, addressing the issue of K-rotation and sliding was the key to decreasing implant-related complications.

The TBW technique, which converts tension on the anterior surface of the patella into compressive force on the articular surface, allowing early mobilization, has become the most widely used surgical approach [4, 23]. However, the frequency of re-operation of the tension band fixation was up to one-third [24]. Moreover, the incidence of complications such as skin irritation and ulceration due to the displacement and rotation of K-wires is relatively high [25, 26].

**Fig. 1:**
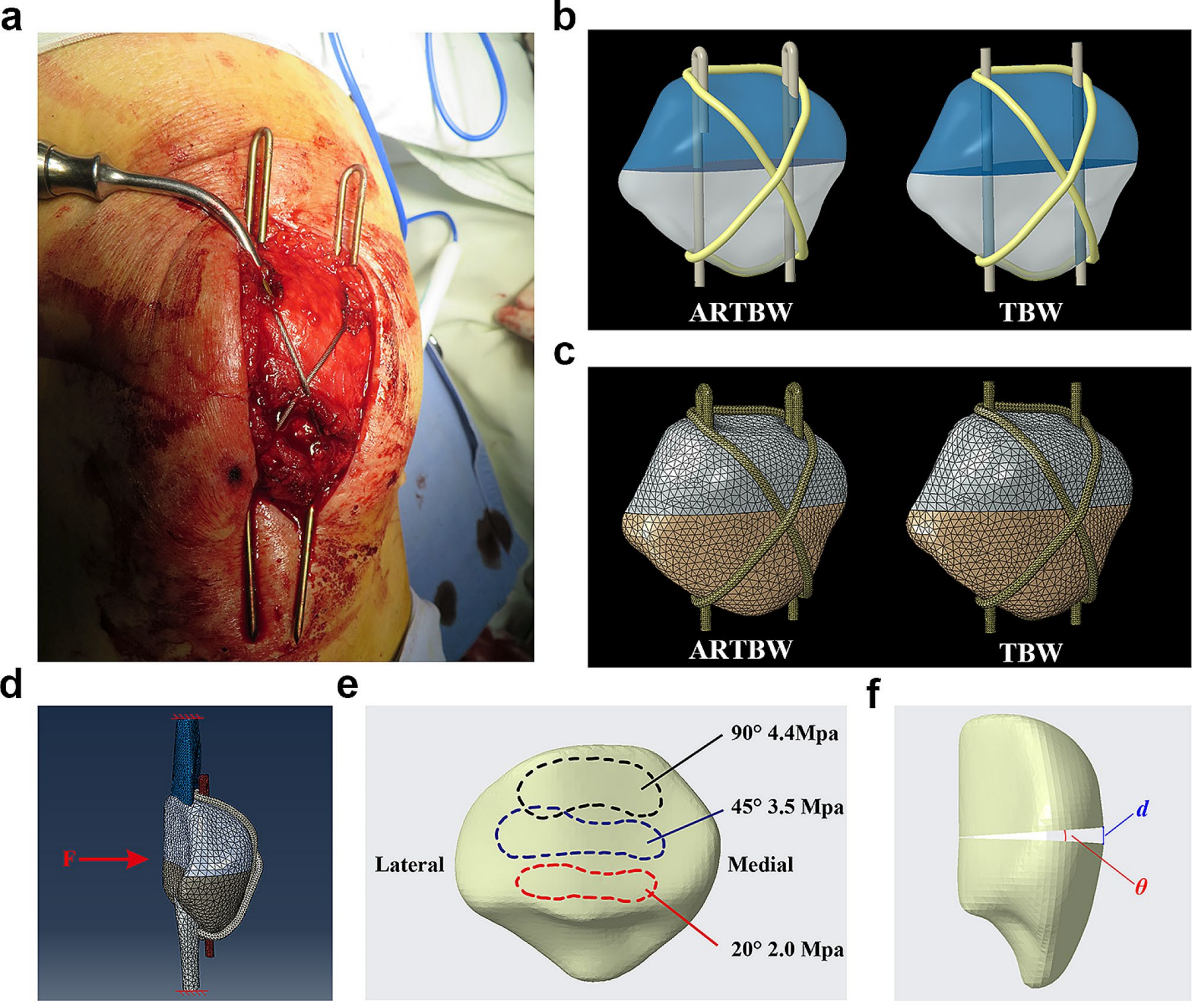
(**a**) The intraoperative image of anti-rotation tension band wiring (ARTBW) technique. (**b**) The 3D geometric model of ARTBW and traditional tension band wiring (TBW). (**C**) The mesh model of ARTBW and traditional TBW. (**d**) The boundary condition and load type (sagittal view). (**e**) The loading site and strength at knee flexion of 20 degrees, 45 degrees, and 90 degrees (posterior view). (**f**) The measured index, the fracture’s maximum displacement gap ($\:\varvec{d}$), and the displacement angle ($\:\varvec{\theta\:}$) after loading in this study (sagittal view)

In order to solve the problem of displacement and rotation of K-wires, we modified the traditional TBW technique and applied it to patellar fracture. We bent the proximal end of the K-wire and implanted it into the patella body, using the bone of the patella itself to prevent the rotation and displacement of the K-wires (Fig. 1a). This new modified TBW technique was called ARTBW. In our previous study, we compared the surgical outcomes of traditional TBW and ARTBW, and the clinical results showed that ARTBW presented a satisfactory clinical outcome and could well address the issue of rotation and displacement of K-wires [15]. However, the evidence for ARTBW in biomechanics is inadequate.

This study aimed to compare the biomechanical characteristics of the traditional TBW and the ARTBW and validate the mechanical stability and anti-rotation effectiveness of the new technique by finite element analysis and mechanical testing.

## Materials and methods

### FEA

The patella geometry in this study was obtained from a computer tomography (CT) image of the left knee of a healthy 26-year-old male volunteer with a height of 182 centimeters and a weight of 75 kg. The CT scanning parameters and post-processing process could be found in Supplementary Material 1. The images were then recorded onto an optical disc in the international standard digital imaging and communications in medicine (DICOM) format for archival purposes.

#### Construction of the 3D model

The DICOM-formatted CT slice images of the knee were imported into MIMICS^®^ 21.0 (Materialize’s Interactive Medical Image Control System/Materialize NV, Belgium). Following noise reduction, the determination of optimal bone tissue boundaries within the threshold range of 226–1691 Hounsfield units, elimination of soft tissue surrounding skeletal images, selective segmentation based on anatomical structures, region-growing, surface gap filling, and smoothing of various patellar components, the geometric model of the patella was successfully constructed (Fig. 1b).

The model was then exported in STL format files, which underwent repair and optimization using the Geomagic Studio (3D Systems Inc., NC, USA). Subsequently, the model surface’s triangular facets were fitted with a smooth and continuous surface, ultimately generating a cohesive surface model. The patellar solid model was imported into the Creo Parametric 10.0 (PTC Inc., USA) in IGES format [27]. By applying a 1 mm inward offset, the cortical and trabecular bone portions of the patellar solid model were separately established [28].

In the Creo Parametric program, we first establish a fracture line on the central horizontal line of the patella to create an AO/OTA 34-C1 type fracture. Subsequently, we created models of the patellar ligament and the quadriceps tendon to simulate the structure of a knee extension mechanism. Then, we constructed models for the K-wire and the tension band, setting the diameter of the K-wire at 2.0 mm and the tension band at 1.8 mm. The proximal end of the K-wires of ARTBW was bent 180°. The simplified model of traditional TBW did not do any bending. Finally, we assemble the models of the patellar fracture, the K-wire, and the tension band. Two K-wire was inserted into the patella in the coronal plane, with the main body of the K-wire placed at a depth of one-half. In the sagittal view, the K-wire’s depth or sagittal placement was centered. In ARTBW models, the bent end of the K-wire was inserted into the patella body, and the insertion depth of the bent proximal K-wire accounted for one-fourth of the total length of the patella. The tension band was wrapped around the K-wires in a figure-eight configuration. Thus, we obtained two models fixed with K-wire and tension bands for the patellar fractures.

#### Mesh generation and material parameters

The resulting models were then imported into the Abaqus/CAE 2020 program (Dassault System Inc., Waltham, MA, USA) for finite element analysis. In this study, the patella, patellar ligament, and quadriceps tendon were all set to be isotropic, homogeneous linear elastic materials. Then, constructing mesh for all solid models in Abaqus (Fig. 1c). The material properties were determined according to previous studies [29–31]. The type of mesh elements, the number of elements for all solid models, and the corresponding material parameters are displayed in Table 1.

**Table 1:** Material properties for components in finite element models

Component	Young’s modulus (MPa)	Poisson’s ratio	Element type	Element number
Cancellous bone	24,100	0.28	C3D10	22,805
Cortical bone	2410	0.20	C3D10	126,706
Quadriceps tendon	500	0.40	C3D10	10,882
Patellar tendon	426	0.40	C3D10	5109
Kirschner wire (Titanium alloy)	110,000	0.30	C3D10	28,463
Tension band (Titanium alloy)	110,000	0.30	C3D10	7142

#### Boundary conditions and loads

Cortical bone was set in bonded contact with cancellous bone, while the internal fixation device was placed in contact with the patella with a friction coefficient of 0.2. The fracture fragments were also set in contact with each other with a friction coefficient of 0.45. The friction coefficient between K-wires and tension was set to 0.1 [27]. Full constraints were applied to the proximal end of the quadriceps tendon and the distal end of the patellar tendon, with the force applied to the patellar surface (Fig. 1d). When the knee joint was flexed at angles of 20°, 45°, and 90°, the force on the lower, middle, and upper thirds of the patellofemoral joint surface was applied at 2.0, 3.5, and 4.4 MPa, respectively [32, 33] (Fig. 1e).

We measured the maximum von Mises stress of the internal fixation (K-wires and tension band) and the articular surface during knee flexion at 20°, 45°, and 90°. Additionally, we assessed the fracture’s maximum displacement gap ($\:d$), the displacement angle ($\:\theta\:$) after loading, and the rotational angle of the two K-wires under two surgical approaches and loading conditions (Fig. 1f).

### Biomechanical experiments

**Fig. 2:**
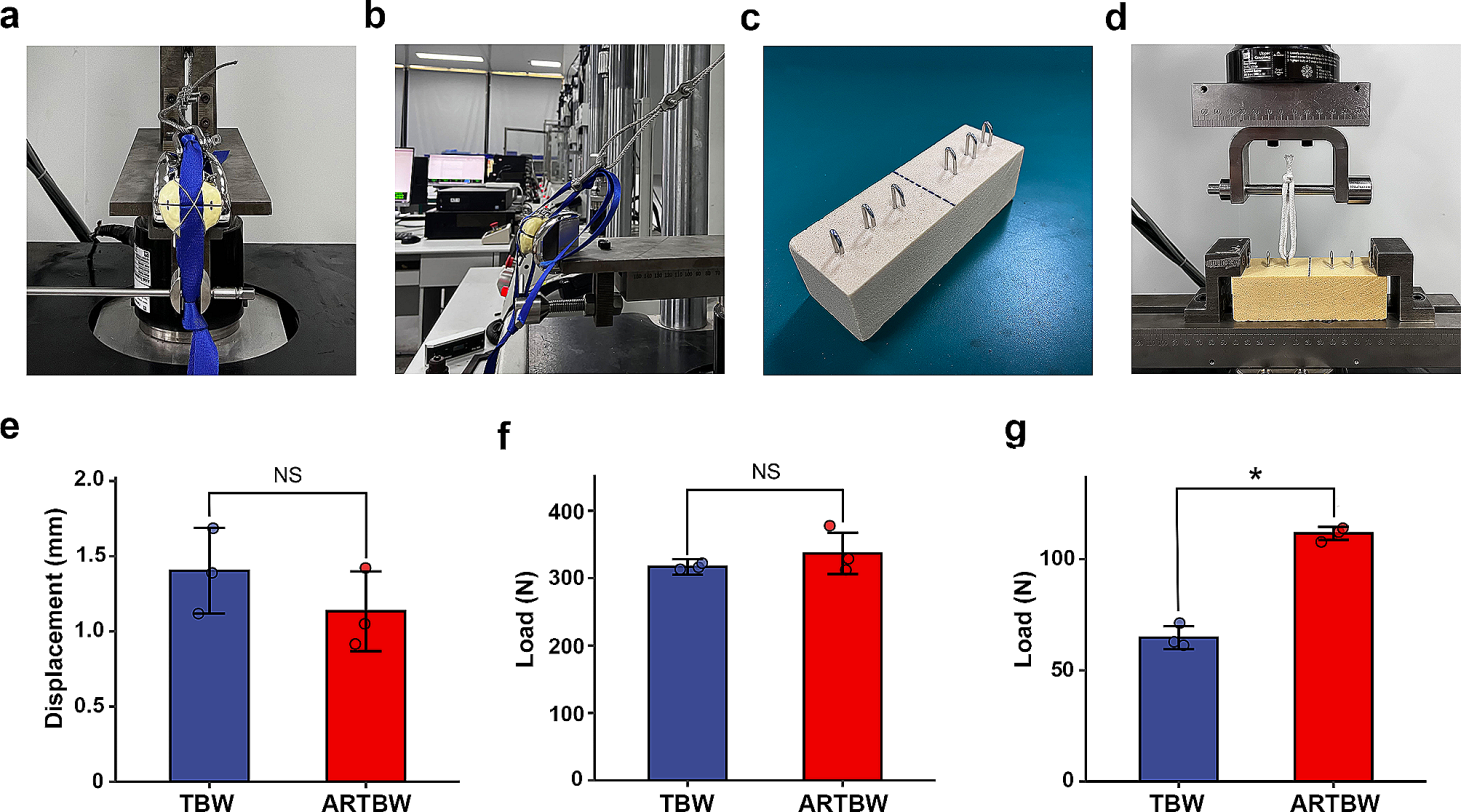
**a-b.** The extensor mechanism model and mechanical testing setting. **c.** The K-wire pull-out experiment model (*n* = 3 for each group) **d.** The K-wire pull-out testing device and setting. **e.** The fracture displacement distance of anti-rotation tension band wiring (ARTBW) and tension band wiring (TBW) after dynamic tensile testing. **f.** The loading value on the testing model until fracture displacement reaches to 2 mm. **g.** The failure pull-out load of ARTBW and traditional TBW (*p* < 0.05)

The mechanical texting used synthetic bone (SYNBONE 1600, Switzerland) with two nylon straps threaded through the fracture line to simulate the quadriceps and patellar tendon (Fig. 2a). The titanium K-wires and steel wire were used to fix the patella fracture. Twelve patellar models were cut transversally at the center of the patella with a line saw to simulate the patellar transverse fracture. Then they were randomly assigned to two groups. One group was fixed with the traditional TBW, whereas the other group employed the modified ARTBW.

Bionix^®^ Tabletop Test Systems (MTS Systems, USA) was used to carry out the biomechanical test. The distal end of the extensor mechanism (patellar tendon) was fixed, and the other end was attached to the steel cable. The pulley on the test machine was adjusted so that the cable was positioned at a 45-degree from the floor (Fig. 2b).

To compare the mechanical performance between the two groups, we conducted static tensile tests and dynamic fatigue experiments. In these tests, the testing machine initially applied a preload of 50 N to the extensor mechanism, and the fracture gap at both ends under 50 N was measured. After machine zero adjusting, an axial load was applied. In the static tensile test, the fracture gap was measured using a vernier caliper at each 20 N increment until the change in the fracture gap reached 2 mm compared to the initial gap. In the dynamic tensile tests, a cyclic load of 100–250 N at a rate of 200 mm/min was then applied to the testing models for 50 cycles. We collected the fracture displacement after 50 cycles under 100 N [34].

Moreover, to test the pull-out strength of different K-wire insertion methods, a K-wire pull-out experiment was conducted. In this experiment, two types of K-wires were implanted into the cancellous bone block with 20 PCF (Sawbones 1522 − 315, USA) (Fig. 2c). The synthetic bone material properties were consistent with previous studies [35, 36]. For the traditional TBW group, the K-wires were directly drilled in, while for the ARTBW group, the wires were bent at the proximal end after drilling and then hammered into the cancellous bone model. A strong rope was hooked onto the bent part of the K-wire’s proximal end, and an upward load was applied at a rate of 10 mm/min, with data collected at a rate of 100 Hz (Fig. 2d). The force at the onset of K-wire extraction from the cancellous bone block was recorded.

### Statistics analysis

The biomechanical data were presented as mean ± standard deviation (SD). For the statistical analysis, the Man Whitney-U test was conducted with *P < 0.05 considered statistically significant. Statistical analyses were carried out utilizing SPSS 27 (IBM, USA).

## Results

**Fig. 3:**
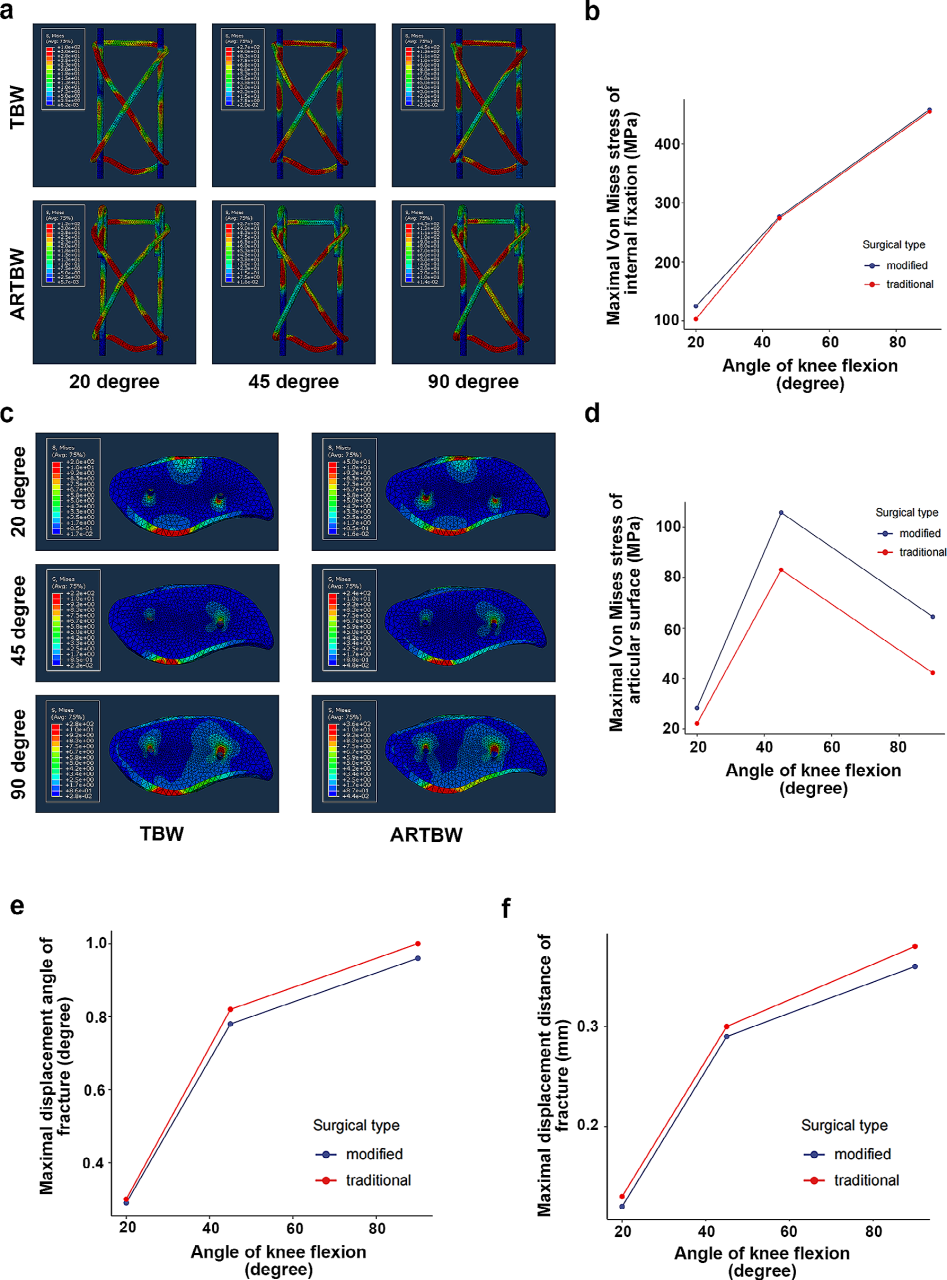
** (a)** The stress distribution on internal fixation of anti-rotation tension band wiring (ARTBW) and traditional tension band wiring (TBW) at knee flexion angles of 20, and 45,90 degrees. **(b)** The quantitative result of the maximum Von Mises stress of internal fixation of traditional TBW and ARTBW. **(c)** The stress distribution on the articular surface of ARTBW and traditional TBW at knee flexion angles of 20, and 45,90 degrees. **(d)** The quantitative result of the maximum Von Mises stress on the articular surface of traditional TBW and ARTBW. **e-f.** The maximal displacement distance and angle of fracture of traditional TBW and ARTBW.

Figure 3a and b demonstrated that the maximum Von Mises stress of internal fixation in both surgical techniques was increased as the angle of knee flexion increased, and the ARTBW was similar to traditional TBW. Figure 3b showed the compression force on the articular surface at different knee flexion angles. The maximum compression force on the articular surface was at a knee flexion angle of 45°, and ARTBW was 28.11%, 27.32%, and 52.86% higher than traditional TBW at knee flexion angles of 20°, 45°, and 90°, respectively. (Fig. 3c; Table 2). As depicted in Table 1; Fig. 3e and f, under the same loading and boundary conditions, the fracture gap and angle in ARTBW were slightly smaller than those in the traditional TBW.

**Table 2:** Comparison of FEA results of traditional tension band wiring (TBW) and anti-rotation tension band wiring (ARTBW).

Results		TBW			ARTBW		
		20 degrees	45 degrees	90 degrees	20 degrees	45 degrees	90 degrees
The maximal Von Mises stress of internal fixation (MPa)		103.03	274.21	454.94	124.61	276.68	458.32
The maximal compressive stress of fracture articular surface (MPa)		22.06	83.03	42.15	28.26	105.72	64.43
The maximal displacement distance of fracture (mm)		0.13	0.30	0.38	0.12	0.29	0.36
The maximal displacement angle of fracture (degree)		0.30	0.82	1.00	0.29	0.78	0.96
The maximal rotation angle of K-wires (degree)	Medial	-0.57	-1.78	-2.29	0.0	0.0	0.0
	Lateral	0.52	1.72	2.41	0.0	0.0	0.0

After loading, the K-wire in the traditional TBW group was rotating, whereas the ARTBW group did not exhibit such a phenomenon. Additionally, we found the rotation angle of K-wires increased as the knee flexion angle increased, with the rotation direction of the wires on both sides being symmetrical and the rotation angles essentially identical (Table 1). The medial K-wire exhibited counterclockwise rotation, while the lateral wire underwent clockwise rotation.

In mechanical testing, the final fracture displacement of traditional TBW(1.402 ± 0.233 mm) was higher than ARTBW (1.117 ± 0.551 mm), and the failure load in ARTBW (336.67 ± 24.49 N) was greater than traditional TBW group (316.67 ± 9.43 N). However, the experimental results indicated that there were no statistically significant differences between the two groups in both static and dynamic tensile tests (Fig. 2e and f, and Table 3). In the K-wire pull-out test, there was a statistically significant difference between the traditional and modified groups(*P* < 0.001), with the ARTBW group exhibiting a significantly greater resistance to pull-out than the traditional group (64.71 ± 4.22 N vs. 111.58 ± 2.38 N) (Fig. 2g; Table 3). This suggests that under the same conditions, the risk of displacement for K-wires in the ARTBW is lower compared to the traditional TBW.

**Table 3:** Comparison of mechanical results of traditional tension band wiring (TBW) and anti-rotation tension band wiring (ARTBW).

Results	Traditional TBW	ARTBW	*P*-value
The fracture displacement distance in dynamic tensile testing (mm, $\:\stackrel{-}{x}\:\pm\:SD$)	316.67$\:\pm\:$9.43	336.67$\:\pm\:$24.94	0.349
The failure loading in static tensile testing (N, $\:\stackrel{-}{x}\:\pm\:SD$)	1.402$\:\pm\:$0.233	1.117$\:\pm\:$0.551	0.538
The pull-out strength (N, $\:\stackrel{-}{x}\:\pm\:SD$)	64.71$\:\pm\:$4.22	111.58$\:\pm\:$2.38	< 0.001*

$\:\stackrel{-}{x}$, mean; SD, Standard Deviation; *P < 0.05

## Discussion

From a biomechanical perspective, the K-wire inserted into the patella had only two degrees of freedom, rotation and sliding. Typically, traditional TBW failed to restrict these two freedoms. The ARTBW involved bending the proximal end of the K-wires by 180° and inserting the bent section into the patellar body as a result restricting the rotation freedom of the K-wire.

The results of FEA revealed that under identical loading conditions, there were no significant differences between the two surgical techniques regarding the maximum fracture gap, angle, and maximum Von Mises stress on implants. Additionally, the FEA results showed the ARTBW had higher compression force on the articular surface. In the mechanical experiment, we chose to simulate 45° of knee flexion for loading, and the mechanical results were consistent with the trend of the finite element results. This demonstrated that the results of FEA and mechanical testing were supporting each other. Furthermore, in the mechanical test, the pull-out strength of the k-wire in ARTBW was significantly higher than in traditional TBW. These results illustrated that ARTBW was more conducive to promoting the healing of patellar fractures and prevented the displacement of K-wires.

Moreover, regarding the rotation of K-wires, in the traditional TBW group, this study observed an increase in the rotation angle with the increase in knee flexion angle. Within the finite element model established in this study, friction existed between the patella and K-wires, the K-wires, and the tension band. So, we inferred that when the patellar fracture was fixed by TBW, the tension on the patellar anterior surface flexion partly converted to the tension on implants. With the action of friction, the tension in the tension band led to the rotation of the K-wires. The ARTBW did not experience any rotation at knee flexion angles of 20°, 45°, and 90°, which was consistent with our expectations.

This study adopted the controlled variable method, with the bending method of the proximal end of the K-wire as the sole variable. We applied the FEA and mechanical testing to investigate the biomechanical characteristics between traditional TBW and ARTBW under the same boundary and load conditions. Compared with previous biomechanical studies of patellar fractures treated by TBW technique, most studies only investigate the biomechanical performance under just one knee flexion angle [11, 37, 38]. The advantage of this study was to compare the biomechanical characteristics between ARTBW and traditional TBW under 20°, 45° and 90°. This allowed for a more comprehensive comparison of these two tension band techniques. Furthermore, compared with previous relevant biomechanical studies, numerical simulation, and mechanical experimental verification in this study made the conclusion more convincing.

This study had some limitations which need to be considered. Firstly, this study employed finite element analysis and mechanical testing methods, simplifying the finite element and mechanical testing model without considering the impact of soft tissues within the knee joint on the patella. Secondly, the finite element models established in this study were treated as isotropic, homogeneous, linear elastic materials.

## Conclusions

In summary, (1) the ARTBW retained the advantages of the traditional TBW and increased the compressive force on the joint surface, thereby promoting fracture healing. (2) The ARTBW effectively prevented the K-wire rotation and increased the pull-out strength of the K-wire, which could reduce the complication caused by K-wire rotation and sliding. Combined with our previous clinical study outcome of ARTBW, the novel ARTBW may be a superior surgical approach for transverse patellar fractures.

### Electronic supplementary material

Below is the link to the electronic supplementary material.

Supplementary Material 1

## Data Availability

No datasets were generated or analysed during the current study.

## Abbreviations

K-wire: Kirschner wire
TBW: Tension band wiring
ARTBW: Anti-rotation tension band wiring
FEA: Finite element analysis
