# Supplementary material for: An innovative anti-rotation tension band wiring for treating transverse patellar fractures: finite element analysis and mechanical testing
Source: J Orthop Surg Res. 2024 Jul 19;19:416. doi: 10.1186/s13018-024-04902-w (PMC11264867; doi:10.1186/s13018-024-04902-w)
Supplement: Supplementary file 1 — Supplementary Material 1 [file 13018_2024_4902_MOESM1_ESM.docx]

**Supplementary material**

**Supplementary material 1: The CT scanning parameters and post-processing process**

The CT (320 slice, General Electric, Milwaukee, WI, USA) was taken with tube voltage and the current was set to 120 kV and automatic, respectively. The pitch was 1.0, rotation time 0.5 s and collimation 128 × 0.6 mm (mm). The slice thickness was 5 mm and a resolution of 512×512 pixels. Post-processing involved interpolation and magnification of the raw data on the machine, resulting in continuous images with the specified slice thickness.
